# *Pseudomonas aeruginosa* Elastase Provides an Escape from Phagocytosis by Degrading the Pulmonary Surfactant Protein-A

**DOI:** 10.1371/journal.pone.0027091

**Published:** 2011-11-01

**Authors:** Zhizhou Kuang, Yonghua Hao, Brent E. Walling, Jayme L. Jeffries, Dennis E. Ohman, Gee W. Lau

**Affiliations:** 1 Department of Pathobiology, University of Illinois at Urbana-Champaign, Urbana, Illinois, United States of America; 2 Department of Microbiology and Immunology, Virginia Commonwealth University School of Medicine, Richmond, Virginia, United States of America; The University of Texas Health Science Center at San Antonio, United States of America

## Abstract

*Pseudomonas aeruginosa* is an opportunistic pathogen that causes both acute pneumonitis in immunocompromised patients and chronic lung infections in individuals with cystic fibrosis and other bronchiectasis. Over 75% of clinical isolates of *P. aeruginosa* secrete elastase B (LasB), an elastolytic metalloproteinase that is encoded by the *lasB* gene. Previously, *in vitro* studies have demonstrated that LasB degrades a number of components in both the innate and adaptive immune systems. These include surfactant proteins, antibacterial peptides, cytokines, chemokines and immunoglobulins. However, the contribution of LasB to lung infection by *P. aeruginosa* and to inactivation of pulmonary innate immunity *in vivo* needs more clarification. In this study, we examined the mechanisms underlying enhanced clearance of the Δ*lasB* mutant in mouse lungs. The Δ*lasB* mutant was attenuated in virulence when compared to the wild-type strain PAO1 during lung infection in SP-A^+/+^ mice. However, the Δ*lasB* mutant was as virulent as PAO1 in the lungs of SP-A^-/-^ mice. Detailed analysis showed that the Δ*lasB* mutant was more susceptible to SP-A-mediated opsonization but not membrane permeabilization. *In vitro* and *in vivo* phagocytosis experiments revealed that SP-A augmented the phagocytosis of Δ*lasB* mutant bacteria more efficiently than the isogenic wild-type PAO1. The Δ*lasB* mutant was found to have a severely reduced ability to degrade SP-A, consequently making it unable to evade opsonization by the collectin during phagocytosis. These results suggest that *P. aeruginosa* LasB protects against SP-A-mediated opsonization by degrading the collectin.

## Introduction

Pulmonary surfactant is a layer of lipoprotein complex with critical surface tension lowering properties, which reduces the work of breathing and helps to maintain airspace patency. Also, it protects the lungs against inhaled air laden with microbes, oxidants, pollutants and allergens [1]–[7]. About 10% of the surfactant layer consists of proteins that have been identified as surfactant protein-A (SP-A), SP-B, SP-C and SP-D. The lung immune defense functions of surfactant are primarily mediated by SP-A and SP-D, which are members of the collectin family of proteins [2], [6], [8]. Severe depletion of SP-A and SP-D has been associated with several respiratory diseases including bacterial pneumonia, adult respiratory distress syndrome, and cystic fibrosis (CF) [9]–[14]. SP-A^-/-^ and SP-D^-/-^ mice have been shown to be more susceptible to lung infection by *P. aeruginosa* and other pathogens [2], [7], [15].

In the past decades, studies have demonstrated that SP-A is an important component of the pulmonary innate immune system. SP-A opsonizes and enhances the phagocytosis of a myriad of microbial pathogens in a calcium-dependent manner [2], [3], [6], [7], [15], [16]. Also, SP-A activates phagocytic cells and upregulates the expression of host cell-surface receptors involved in microbial recognition [8], [17]–[21]. Most recently, we and others have reported that SP-A also directly kills microbes in a macrophage-independent manner by increasing the permeability of microbial membranes [22]–[27]. However, the mechanism by which SP-A permeabilizes microbial membranes and its relative importance in the lung defense is not clear. For example, it is not known whether microbes that are membrane permeabilized by SP-A are phagocytized more efficiently than the microbes with intact cell membranes.

*P. aeruginosa* is a Gram-negative bacterial pathogen that causes both acute pneumonitis in immunocompromised patients and chronic lung infections in individuals with CF and non-CF bronchiectasis, and chronic obstructive pulmonary disease (COPD) [28]–[32]. Multiple virulence factors of *P. aeruginosa* contribute to lung infection [33]. These virulence determinants work in concert either offensively to inactivate components of host immune response, or defensively to camouflage or evade host response [33]. Cell surface associated virulence factors of *P. aeruginosa* include pili, flagella, alginate, and lipopolysaccharides (LPS) [33]. Type III and Type IV secretion effectors are injected into the host cells to modulate host immune response [34]–[37]. Secreted components include exotoxin A, phospholipases, phenazines, rhamnolipids and exoproteases [33]. Among the exoproteases, elastase B (LasB), is a major elastolytic zinc metalloproteinase of 33 kDa encoded by the *lasB* gene of *P. aeruginosa* [38], [39]. Also known as pseudolysin, LasB has received much attention and been recognized as an important virulence factor. LasB is thought to damage host tissues through hydrolysis of the components of extracellular matrix and by breaching the endothelial and epithelial barriers by attacking intercellular tight junctions [40], [41]. Under *in vitro* experimental conditions, LasB degrades numerous components of innate and adaptive immune systems, including SP-A and SP-D [42], [43], cytokines and chemokines TNF-α, IFN-γ, IL-2 and IL-8 [40], [44]–[47], and antibacterial peptide [48]. Also, there are reports of elastase inactivating secretory immunoglobulin A, immunoglobulin G and opsonin C3 [31], [48]–[50]. Most recently, we have confirmed that *P. aeruginosa* LasB is able to degrade lysozyme *in vitro* [22], [51].

Despite numerous *in vitro* studies, direct evidence of LasB-mediated proteolytic activities in the lungs, and to what extent they contribute to the pathogenesis of *P. aeruginosa* requires more investigation. In this study, we compared the virulence role of LasB by using wild-type *P. aeruginosa* strain PAO1 versus an isogenic Δ*lasB* mutant strain in an acute model of lung infection in SP-A^+/+^ versus SP-A^-/-^ mice.

## Results

### The Δ*lasB* bacteria are severely attenuated in exoprotease activities

**Figure 1:**
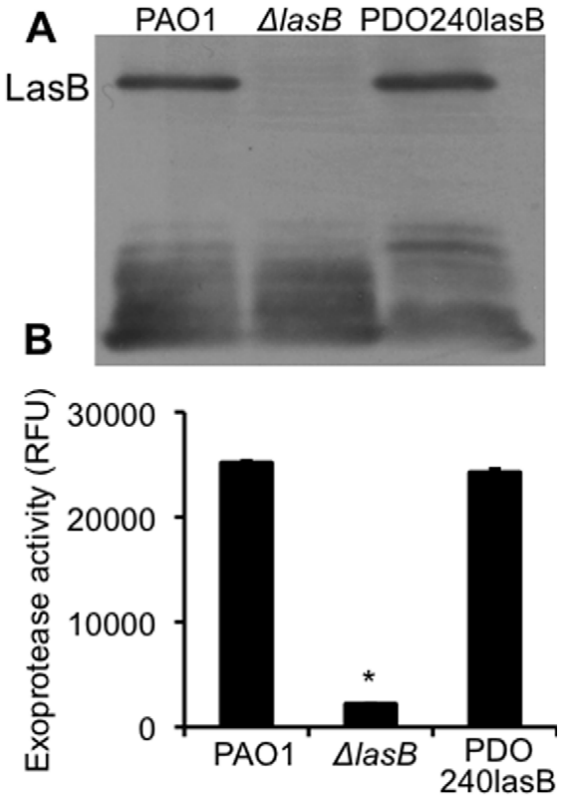
The Δ*lasB* mutant has severely attenuated exoprotease activity. (A) Western blot analysis of the LasB production in the supernatant of PAO1, Δ*lasB* mutant and PDO240LasB as detected by anti-LasB antibody. **(**B) Proteolytic activity of stationary phase culture supernatant collected from PAO1, Δ*lasB* and PDO240lasB. Experiments were performed independently three times in triplicates. The mean + standard deviation from one representative experiment is shown. *p<0.01 when comparing the exoprotease activity of Δ*lasB* against PAO1 or PDO240lasB.

We examined the amounts of LasB in the supernatants of stationary phase *P. aeruginosa* cultures. As expected, the PDO240 mutant (Table 1) (from here in Δ*lasB*) bacteria did not secrete LasB. In contrast, both the wild-type PAO1 and the genetically complemented PDO240LasB bacteria produced the 33 kDa LasB (Figure 1A). In addition, the Δ*lasB* bacteria had approximately 10-fold less total exoprotease activity when compared to PAO1 and PDO240LasB (Figure 1B).

**Table 1:** *P. aeruginosa* strains used in this study.

*Bacterial Strains*	*Relevant characteristics*	Reference
*P. aeruginosa*		
PAO1	Wild-type	M. Vasil, 58
PAO1-gfp	PAO1 strain harboring the green fluorescent protein expression broad host range plasmid pUCP19-gfp	This study
PDO240 (Δ**lasB)	Elastase-deficient mutant derived from PAO1	[57]
Δ*lasB* -gfp	Δ*lasB* mutant harboring GFP plasmid pUCP19-gfp	This study
PDO240lasB	Δ*lasB* mutant harboring a wild-type *lasB* gene on plasmid pKSM3	This study
*E. coli*		
DH5α	*F-f80 DlacZDM15 endA1 recA1 hsdR17 (r^-^km^+^k) supe44 thi-1 l-gyr A96 relA1 D(lacZYA-argF) U169*	[66]
Plasmids		
pUCP19-gfp	Broad host range vector. Polylinker *lacZ*, lac^iq^ selection, *bla, gfp*	[26]
pKSM3	pLAFR3 with *lasB* gene on a 2.6 kb EcoRI-Pstl fragment	[65]

### The Δ*lasB* bacteria are cleared more efficiently following lung infection in SP-A^+/+^ but not SP-A^-/-^ mice

**Figure 2:**
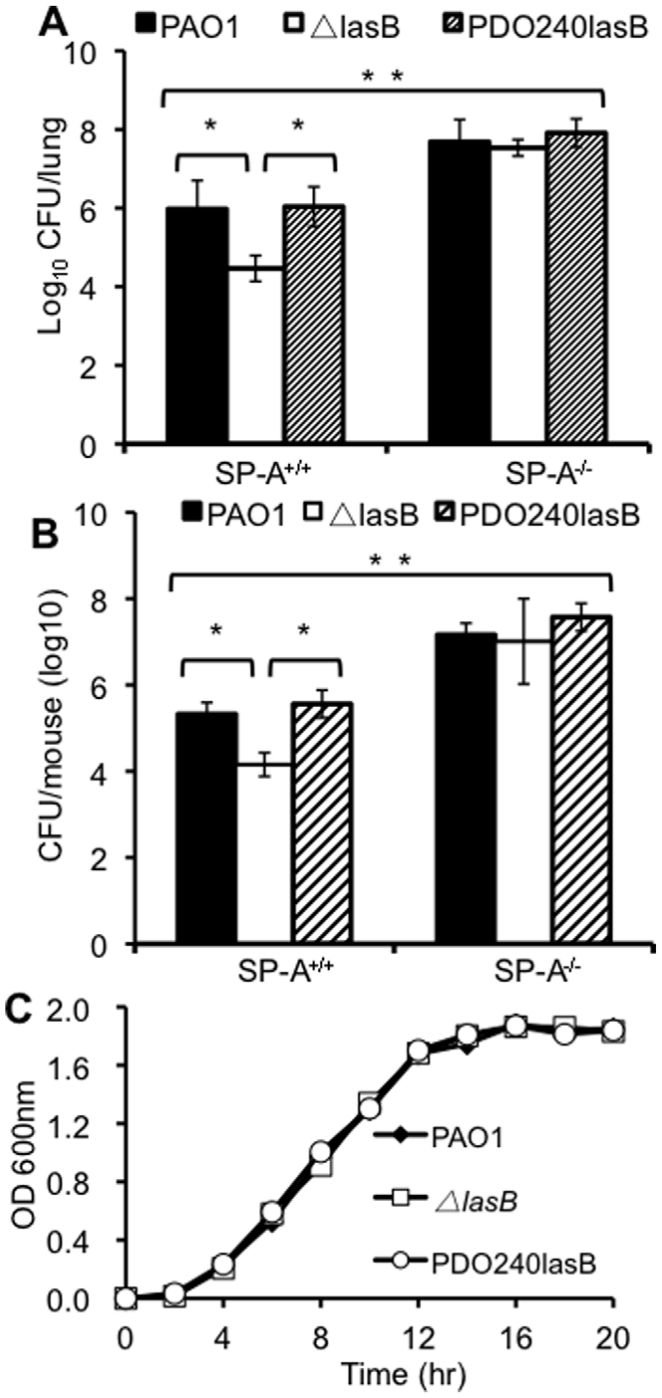
The Δ*lasB* mutant is attenuated for virulence in SP-A^+/+^ mice. (A) Respiratory tract infections with wild-type PAO1, Δ*lasB* mutant or genetically-complemented PDO240lasB bacteria were performed by intranasal inoculation of anesthetized SP-A^+/+^ or SP-A^-/-^ mice. Mouse lungs were harvested 18 hr after infection for CFU enumeration. Data are the mean CFU ± SE (n = 5 per group). * *p*<0.05 when comparing lungs of SP-A^+/+^ mice infected with PAO1 and PDO240lasB versus Δ*lasB*; ** *p*<0.05 when compared between SP-A^+/+^ and SP-A^-/-^ mice infected with PAO1, Δ*lasB* or PDO240lasB bacteria. (B) Mouse lungs were harvested 36 hr after infection for CFU enumeration. Data are the mean CFU ± SE (n = 5 per group). * *p*<0.05 when comparing lungs of SP-A^+/+^ mice infected with PAO1 and PDO240lasB versus Δ*lasB*; ** *p*<0.05 when compared between SP-A^+/+^ and SP-A^-/-^ mice infected with PAO1, Δ*lasB* or PDO240lasB bacteria. (C) Attenuation of Δ*lasB* bacteria in mouse lungs was not due to a slower growth rate. Bacterial growth was assessed by absorbance at OD_600_. The data from one of the three independent experiments are shown.

To determine the contribution of LasB to lung infection, we compared the virulence of the wild-type *P. aeruginosa* PAO1, the isogenic Δ*lasB* mutant, and the genetically-complemented strain PDO240lasB in a mouse acute pneumonia model of single infection studies. In the absence of bacterial infection, histopathological features of SP-A^-/-^ mouse lungs were indistinguishable when compared to the lungs of SP-A^+/+^ mice (data not shown). Eighteen hr after intranasal inoculation with PAO1 or PDO240lasB, SP-A^+/+^ mice showed signs of infection and respiratory distress but were not moribund. In contrast, PAO1-infected SP-A^-/-^ mice were moribund and had to be euthanized (data not shown). The number of viable wild-type PAO1 or PDO240lasB bacteria in SP-A^-/-^ were 1.72 log and 1.88 log higher than in SP-A^+/+^ mice, respectively (Figure 2A). Eighteen hr after infection with the Δ*lasB* mutant bacteria, the lungs of SP-A^+/+^ mice showed little sign of disease. In contrast, SP-A^-/-^ infected with Δ*lasB* mutant bacteria developed significant respiratory distress or were moribund, and had to be euthanized (data not shown). The viable counts of Δ*lasB* mutant were 1.5 log lower than PAO1 in SP-A^+/+^ mice. However, the number of Δ*lasB* bacteria was 3.1 log higher in SP-A^-/-^ mice than in SP-A^+/+^ mice, and was statistically indistinguishable when compared to the number of PAO1 and PDO240lasB bacteria in the SP-A^-/-^ mice (Figure 2A). By 36 hr, the number of bacteria for each strain in the SP-A^+/+^ mice further decreased by approximately 0.5 log. However, the decrease was not obvious in the Sp-A^-/-^ mice (Figure 2B). These results indicate that Δ*lasB* bacteria are more virulent in the lungs of SP-A^-/-^ mice than in the lungs of SP-A^+/+^ mice. Virulence attenuation in Δ**lasB bacteria was not due to reduced growth rate as wild-type PAO1, Δ*lasB* and PDO240LasB bacteria had virtually identical growth kinetics (Figure 2C).

**Figure 3:**
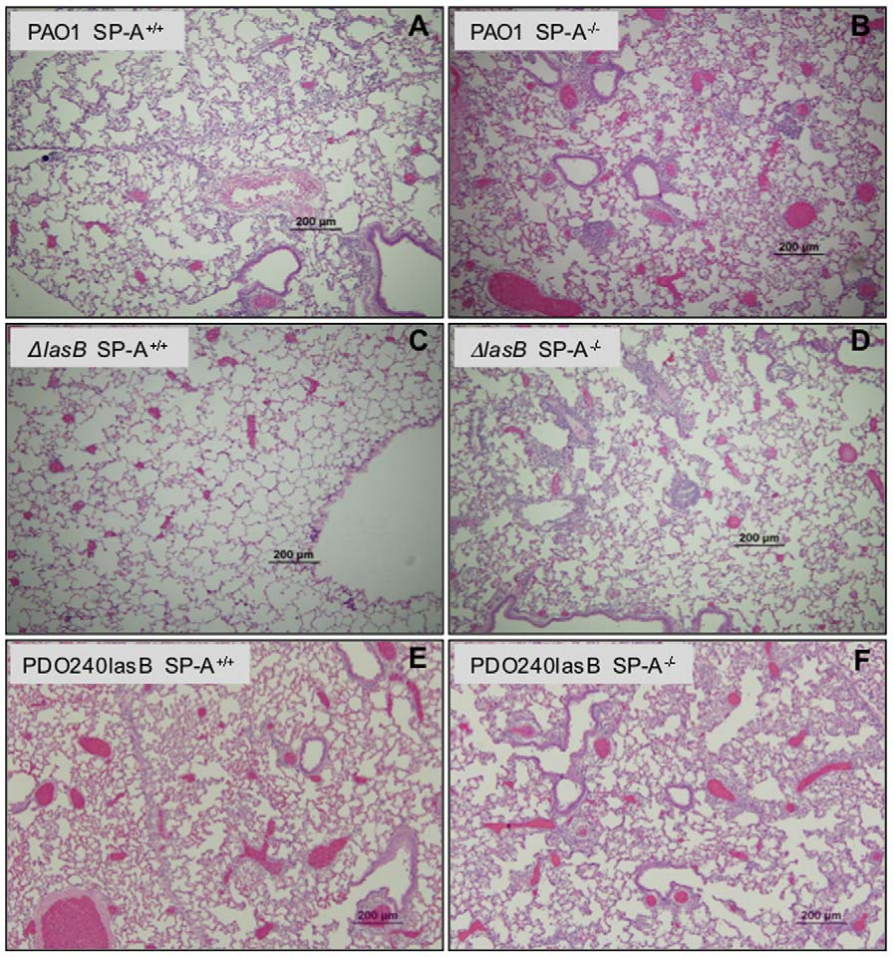
Histopathology of *P. aeruginosa* infected lungs. Representative H&E-stained lung sections from SP-A^+/+^ and SP-A^-/-^ mice (n = 5) 18-hr post intranasal instillation of PAO1 (A-B), Δ*lasB* (C-D) and PDO240lasB (E-F) bacteria.

Next, we examined various infected mouse lungs with histopathological methods (Figure 3). Our analysis showed that PAO1 and PDO240lasB caused more severe alveolitis with pulmonary infiltrates (Figure 3A and 3E) whereas the Δ*lasB* mutant only caused mild alveolitis in the lungs of SP-A^+/+^ mice (Figure 3C). In contrast, PAO1, Δ*lasB* mutant and PDO240lasB caused similar amounts of consolidation with more areas of pneumonia in SP-A^-/-^ lungs (Figure 3B, 3D, and 3F). These results indicate that LasB plays an important protective role against anti-*P. aeruginosa* activity mediated by SP-A.

### The Δ*lasB* bacteria are deficient in their ability to degrade SP-A

**Figure 4:**
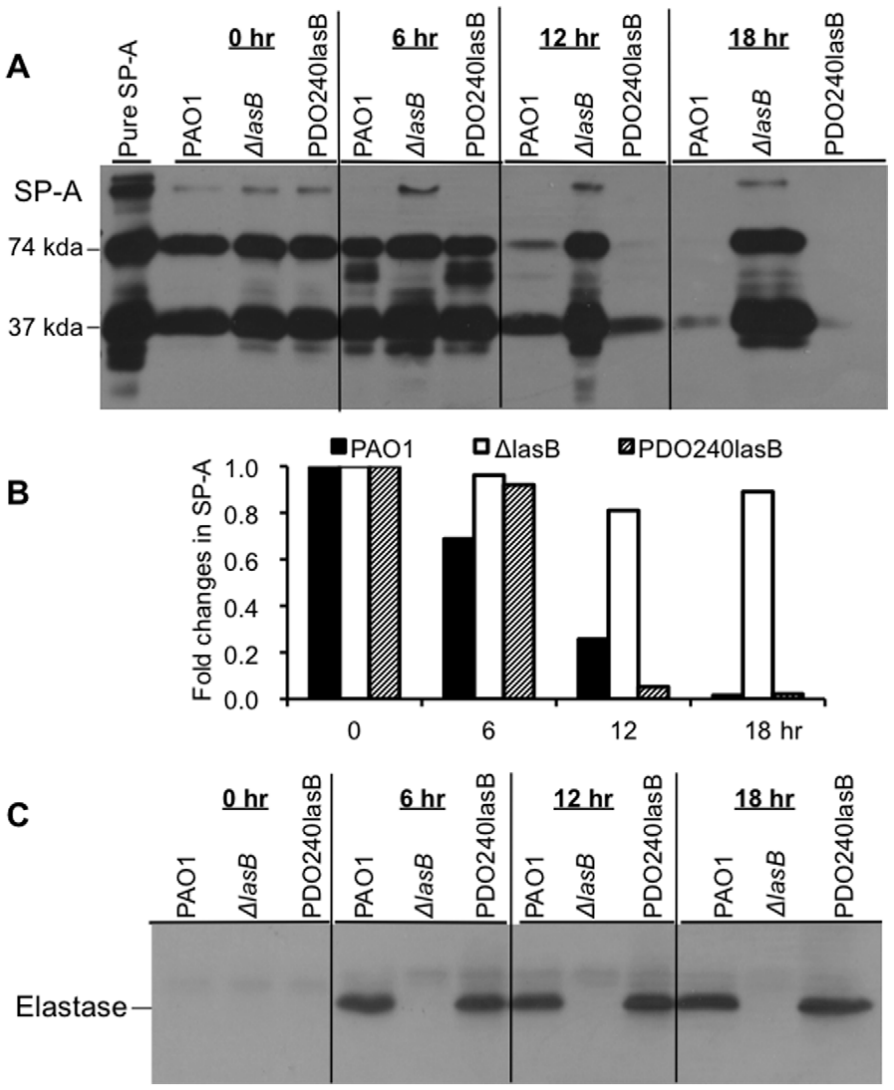
SP-A-degrading ability is reduced inΔ** *lasB* mutant bacteria *in vitro*. (A) hSP-A (25 µg) was incubated with 1×10^8^ PAO1, Δ*lasB* or PDO240LasB bacteria for the indicated time intervals. hSP-A degradation was assessed by western blot analyses using 10 µl of SP-A/bacterial suspension. Image from one of the three independent experiments is shown. (B) Densitometry quantification of hSP-A degradation in A. (C) Production of LasB in the mixture was assessed by western blot analyses using 10 µl of supernatant from the hSP-A/bacterial suspension. Immunoblots were probed with anti-SP-A (A) or anti-LasB (C) antibody, respectively.

Previously, it has been shown that *P. aeruginosa* elastase degrades human SP-A (hSP-A) [22], [43], [52]. Here, we examined the ability of Δ*lasB* mutant on its ability to degrade hSP-A. The hSP-A (25 µg) was incubated with 1×10^8^ wild-type PAO1, Δ*lasB*, or genetically complemented PDO240lasB bacteria (Table 1) for the indicated time intervals. After 6 hr of incubation, the degradation of hSP-A by PAO1 and PDO240lasB bacteria was evident (Figure 4A). By 18 hr post incubation, hSP-A was almost completely degraded by PAO1 and PDO240lasB. In contrast, there was only minimal degradation of hSP-A by the Δ*lasB* bacteria, with majority of the collectin remaining intact even after 18 hr of exposure (Figure 4A). Densitometry analysis indicates that PAO1 degraded approximately 40% hSP-A after 6 hr incubation. By 12 and 18 hr, majority of the hSP-A had been degraded (Figure 4B). Increasing amount of hSP-A degradation was correlated with higher amount of LasB secretion by PAO1 and PDO240lasB as the time of incubation was lengthened (Figure 4C). The degradation of SP-A was not influenced by the presence or absence of Zn^2+^, suggesting that LB provided sufficient Zn^2+^ for the proteolytic activities of LasB (Figure S1). These results suggest that Δ*lasB* bacteria are strongly attenuated in their ability to degrade hSP-A, and that LasB is a major exoprotease of *P. aeruginosa* that is responsible for the removal of hSP-A.

### The Δ*lasB* bacteria are impaired in the degradation of SP-A during infection of SP-A^+/+^ mouse lungs

**Figure 5:**
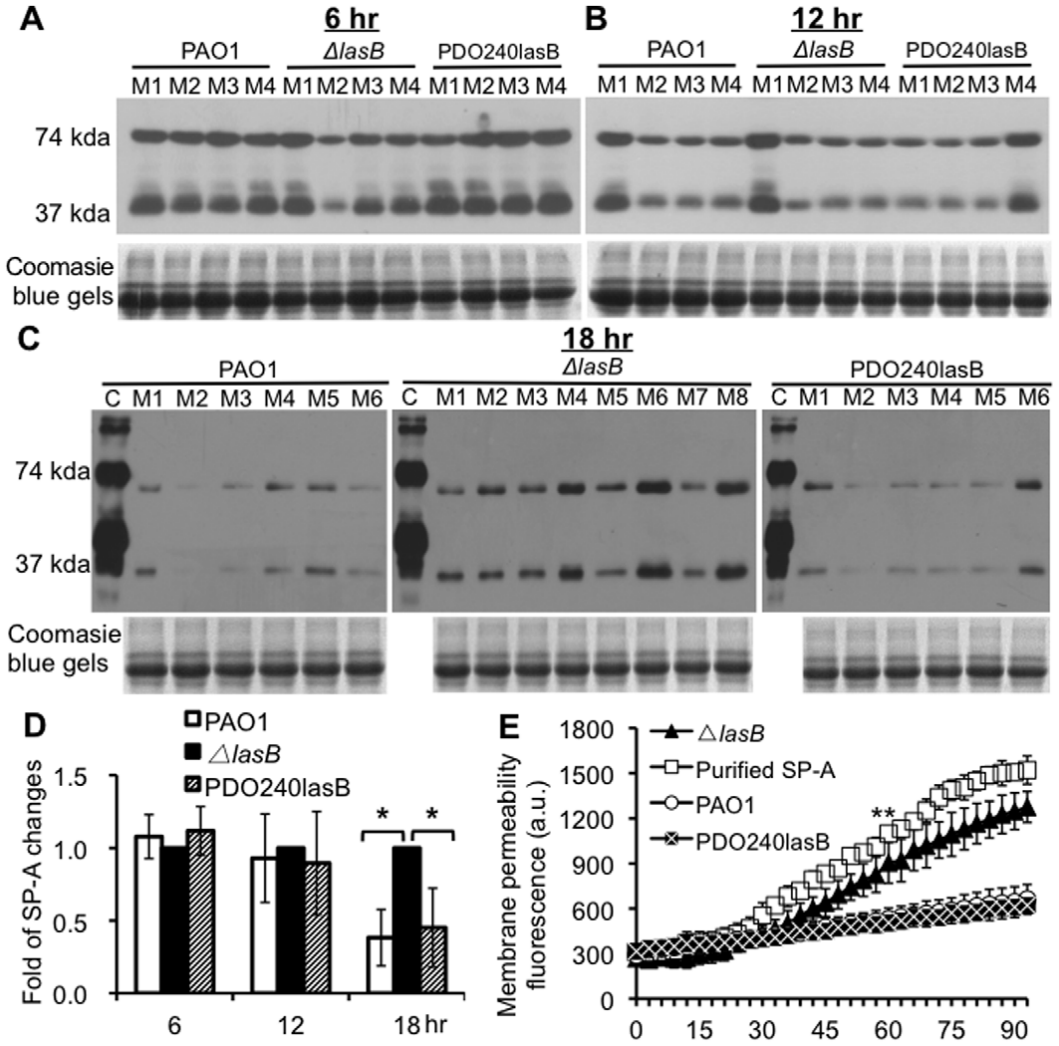
Elastase deficient Δ*lasB* mutant is attenuated in the degradation of SP-A during lung infection. (A-C) The amounts of intact mSP-A were not visibly changed at 6-hr (A) or 12- hr (B) post-infection. By 18 hr post-infection (C), intact mSP-A was reduced in the BAL fluid from PAO1- or PDO240lasB-infected SP-A^+/+^ mice (n = 6), suggesting that mSP-A was degraded in mouse lungs. In contrast, more abundant mSP-A was clearly visible in the BAL fluids from Δ*lasB* (n = 8). C  =  Purified human SP-A. M1 – M8  =  BAL of mice infected with *P. aeruginosa*. Western blot analyses were performed using a polyclonal antibody against SP-A. (D) Densitometry analysis of mSP-A degradation by PAO1, Δ*lasB* and PDO240lasB in mouse lungs. The amounts of remaining mSP-A in Δ*lasB* were set to the value of 100%. *p<0.05 when compared the amount of mSP-A in BAL fluids from lungs infected with PAO1 or PDO240lasB against BAL fluids from Δ*lasB*-infected mice. (E) Mouse BAL from Δ*lasB*-infected animals contains intact mSP-A that permeabilizes bacterial membranes. Pooled BAL fluids (from C) (50 µg/ml total proteins) were used for membrane permeabilization assays. hSP-A (50 µg/ml) was used as positive control. BAL fluids from PAO1 and PDO240lasB infected mice failed to permeabilize *E. coli* membranes. hSP-A and BAL samples from Δ*lasB*-infected mice were able to permeabilize bacterial membranes of *E. coli* DH5α at higher levels. Experiments were performed independently three times in triplicates. The mean + standard deviation from one representative experiment is shown. **p<0.05 from 60 min onward when comparing the membrane permeabilization of *E. coli* by pure SP-A or BAL samples from Δ*lasB*-infected mice against BAL samples from PAO1 or PDO240lasB-infected mice.

Although *in vitro* studies have shown that *P. aeruginosa* secretes elastase to degrade SP-A [22], [43], [52], the biological importance of SP-A removal by LasB and the resulting resistance to clearance during infection of SP-A^+/+^ lungs have not been investigated. We compared the *in vivo* degradation of mouse SP-A (mSP-A) by wild-type PAO1, the elastase-deficient mutant Δ*lasB*, and the complemented strain PDO240lasB in SP-A^+/+^ mice. The amounts of mSP-A in the BAL fluids from mice infected with all three bacterial strains were similar at 6 hr (Figure 5A) and 12 hr post-infection (Figure 5B). However, by 18 hr post-infection, PAO1 or PDO240lasB had significantly lower amounts of mSP-A. In contrast, significant amounts of intact mSP-A dimers and monomers were detected in the BAL of mice infected with Δ*lasB* bacteria (Figure 5C). Western blot data were confirmed by densitometry analyses, which showed that both PAO1 and PDO240lasB degrade significantly more mSP-A than Δ*lasB* mutant (Figure 5D). These results suggest that LasB plays important role in removal of mSP-A *in vivo*.

A previous study has suggested that mSP-A is a principal factor that permeabilizes microbial membranes in the alveolar lining fluid of mouse lungs [53]. Thus, proteolytic degradation of mSP-A by LasB-secreting PAO1 or PDO240lasB would inactivate the ability of mSP-A within lung BAL fluids to permeabilize microbial membranes. To further assess the function of LasB against mSP-A *in vivo*, we compared the ability of BAL fluids from 18 hr post-infection (from Figure 5C) to permeabilize the membrane of *E. coli* DH5α. Purified hSP-A was used as a positive control. Pure hSP-A has the highest levels of membrane permeabilization activity, which was 2.3 and 2.5 fold higher than BAL fluids from PAO1 and PDO240lasB infected SP-A^+/+^ mice after 90 min of incubation (Figure 5E). In contrast, even though the extent of membrane permeabilization on DH5α mediated by pure hSP-A was consistently higher than BAL fluids from Δ*lasB*-infected animals, the difference was not statistically significant (Figure 5E). Importantly, BAL fluids from PAO1 or PDO240lasB *-*infected SP-A^+/+^ mouse lungs, where mSP-A had been degraded by LasB, showed lower ability to permeabilize DH5α (Figure 5Ε). On the contrary, BAL fluids from Δ*lasB*-infected mice permeabilized DH5α bacteria at 1.9 and 2.1 fold higher than BAL from PAO1 and PDO240lasB, respectively, after 90 min of exposure (Figure 5E). Taken together, these results suggest that during infection of mouse lungs, *P. aeruginosa* protects itself against the antimicrobial activities of mSP-A by degrading the collectin through the secretion of LasB.

### The Δ*lasB* bacteria are not susceptible to SP-A-mediated membrane permeabilization

**Figure 6:**
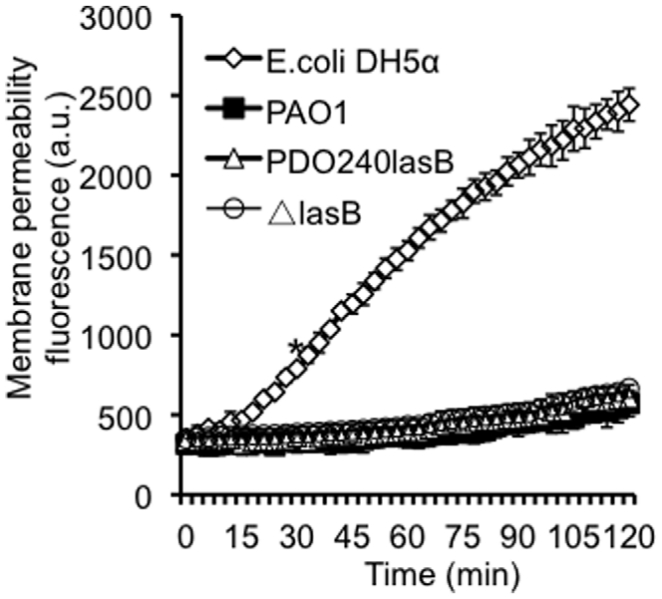
Δ*lasB* mutant bacteria are resistant to SP-A-mediated membrane permeabilization. Membrane permeabilization assays were performed with 1×10^8^ of *E. coli* DH5α or *P. aeruginosa* exposed to hSP-A (50 µg/ml) for 120 min. Three independent experiments were performed in triplicates. The mean + standard deviation from one representative experiment is shown. The membrane permeabilization activity of hSP-A against PAO1, Δ*lasB* and PDO240lasB was not statistically different among all three *P. aeruginosa* strains. *p<0.05 from 35 min onward when comparing the membrane permeabilization of DH5α against PAO1, Δ*lasB* and PDO240lasB.

Previous studies have demonstrated that SP-A protects lungs against microbial infection by opsonization [2], [3], [7], [15]. More recently, we and others have shown that SP-A is capable of directly killing microbes in a macrophage-independent manner, by permeabilizing microbial membranes [22]–[27]. We examined which defense mechanism(s) led to enhanced clearance of Δ*lasB* bacteria in the lungs of SP-A^+/+^ mice. Previously, we have reported that the wild-type *P. aeruginosa* strain PAO1 is resistant to hSP-A-mediated membrane permeabilization [22], [26], [27]. First, we compared the susceptibility of the Δ*lasB* mutant to hSP-A-mediated membrane permeabilization against its parental wild-type PAO1 and the complemented strain PDO240lasB. *E. coli* DH5αcells incubated with hSP-A were used as positive control. As expected, hSP-A permeabilized the membrane of DH5α cells (Figure 6). In contrast, PAO1, Δ*lasB*, and PDO240lasB bacteria demonstrated similar levels of resistance to hSP-A-mediated membrane permeabilization (Figure 6). These results suggest that mSP-A-mediated membrane permeabilization is not responsible for the enhanced clearance of Δ*lasB* bacteria in mouse lungs.

### The Δ*lasB* bacteria are unable to degrade SP-A and are more susceptible to SP-A-mediated opsonization *in vitro*

**Figure 7:**
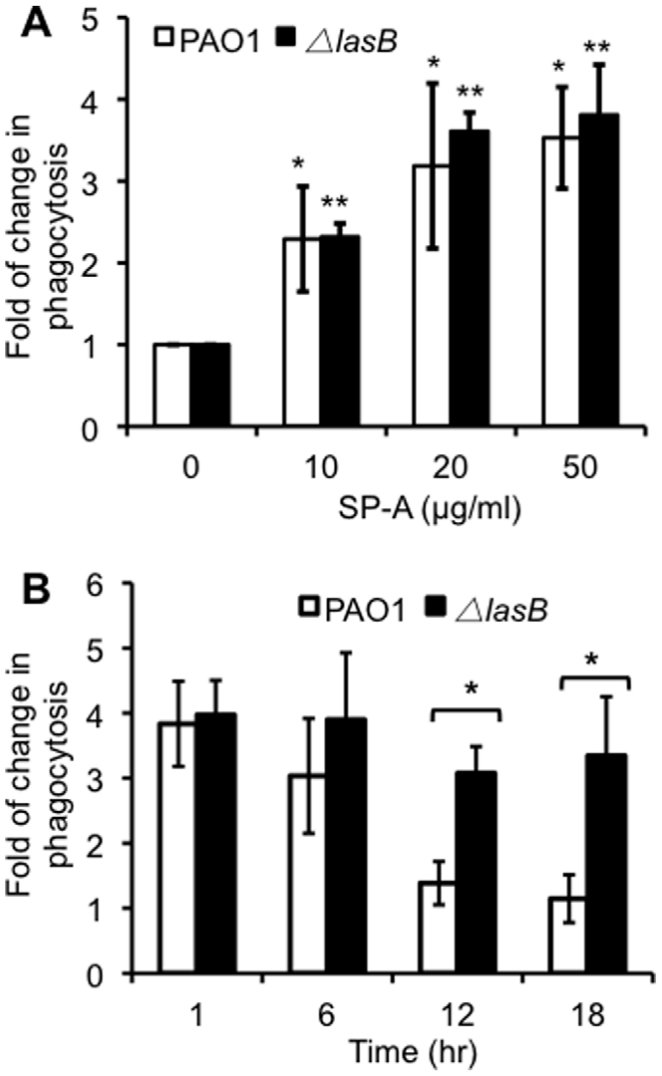
The Δ*lasB* mutant is unable to degrade and impede SP-A-mediated opsonization *in vitro*. *(*A) hSP-A opsonized and increased the phagocytosis of both wild-type PAO1 and Δ*lasB* bacteria in a concentration dependent manner. ∼1×10^7^ PAO1 or Δ*lasB* bacteria were treated with PBS alone or with increasing concentrations of hSP-A for 1 hr in the presence of 1×10^6^ cultured RAW 264.7 macrophages. The number of phagocytized bacteria was determined by gentamicin exclusion assay. The fold increase in phagocytosis was calculated based on the number of engulfed bacteria in macrophages treated with hSP-A versus PBS alone. Three independent experiments were performed in triplicates. The mean + standard deviation from one representative experiment is shown. *^,^ **p<0.01 when comparing the internalized PAO1 or Δ*lasB* mutant pretreated with various concentrations of hSP-A versus PBS alone. (B) The Δ*lasB* mutant bacteria are more susceptible to hSP-A-mediated opsonization. hSP-A (20 µg/ml) was incubated with 1×10^7^ PAO1 or Δ*lasB* bacteria for 1, 6, 12, or 18 hr. At indicated time intervals, the bacteria-hSP-A mixture was added to 1×10^6^ cultured RAW 264.7 macrophages, and incubated for another 1 hr. The number of engulfed bacteria was examined as in (A), and normalized against PAO1 or Δ*lasB* bacteria phagocytized in the absence of hSP-A. Three independent experiments were performed in triplicates. The mean + standard deviation from one representative experiment is shown. *p<0.01 when comparing the number of phagocitized Δ*lasB* bacteria against internalized PAO1 bacteria.

Previously, *P. aeruginosa* has been shown to be susceptible to SP-A-mediated opsonization [54], [55]. Because the Δ*lasB* mutant bacteria were not sensitive to hSP-A-mediated membrane permeabilization, we examined whether they were more susceptible to hSP-A-mediated opsonization. Bacterial phagocytosis assays were performed using the murine macrophages RAW 264.7. The number of *P. aeruginosa* cells internalized by macrophages RAW 264.7 was enumerated by gentamicin exclusion assays [56]. The presence of hSP-A significantly increased the phagocytosis of both the wild-type PAO1 and Δ*lasB* mutant by macrophages in a concentration dependent manner (Figure 7A). When exposed to 10, 20, or 50 µg/ml of hSP-A, the number of Δ*lasB* bacteria internalized by macrophages was 2.3, 3.6 and 3.8 fold higher respectively compared to Δ*lasB* without hSP-A treatment in 60 min. However, the increase in the phagocytosis of Δ*lasB* bacteria was statistically indistinguishable from PAO1. These results suggest that the ability of PAO1 bacteria to secrete LasB does not significantly interfere with the ability of hSP-A to opsonize the bacteria within the short duration (1 hr) under our *in vitro* experimental conditions. This observation is not surprising because a large amount of intact hSP-A still remained after 6 hr of exposure to PAO1, partly due to high amounts of hSP-A (25 µg) used in the experiments (Figure 4).

Next, we examined the impact of prolonged exposure of hSP-A to PAO1 or Δ*lasB* on the ability of the collectin to opsonize the bacteria. hSP-A (20 µg/ml) was preincubated with PAO1 or Δ*lasB* for 1, 6, 12 or 18 hr before the mixture was added to the macrophages for phagocytosis assays. After 60 min of phagocytosis, internalized bacteria were enumerated by gentamicin exclusion assay. The number of internalized PAO1 decreased gradually in a time-dependent manner (Figure 7B), in an inverse relationship to the degradation of hSP-A (Figure 4). By 12 and 18-hr, the number of PAO1 bacteria internalized by macrophages was 1.7-fold and 2.2-fold lower than the Δ*lasB* bacteria, respectively (Figure 7B). As expected, because of its greatly reduced ability to degrade hSP-A, the phagocytosis rate of Δ*lasB* bacteria remained nearly constant throughout the entire experiment. Even though there was a slight decrease in the number of Δ*lasB* bacteria internalized by macrophages exposed to the bacteria/hSP-A mixture from the 12^th^ and 18^th^-hr, the decrease was not statistically significant (Figure 7B). These results are consistent with the observation that Δ*lasB* bacteria lack the ability to degrade hSP-A, and are subsequently opsonized by the collectin and phagocytized by macrophages.

### The Δ*lasB* mutant bacteria are more susceptible to mSP-A mediated opsonization *in vivo*

**Figure 8:**
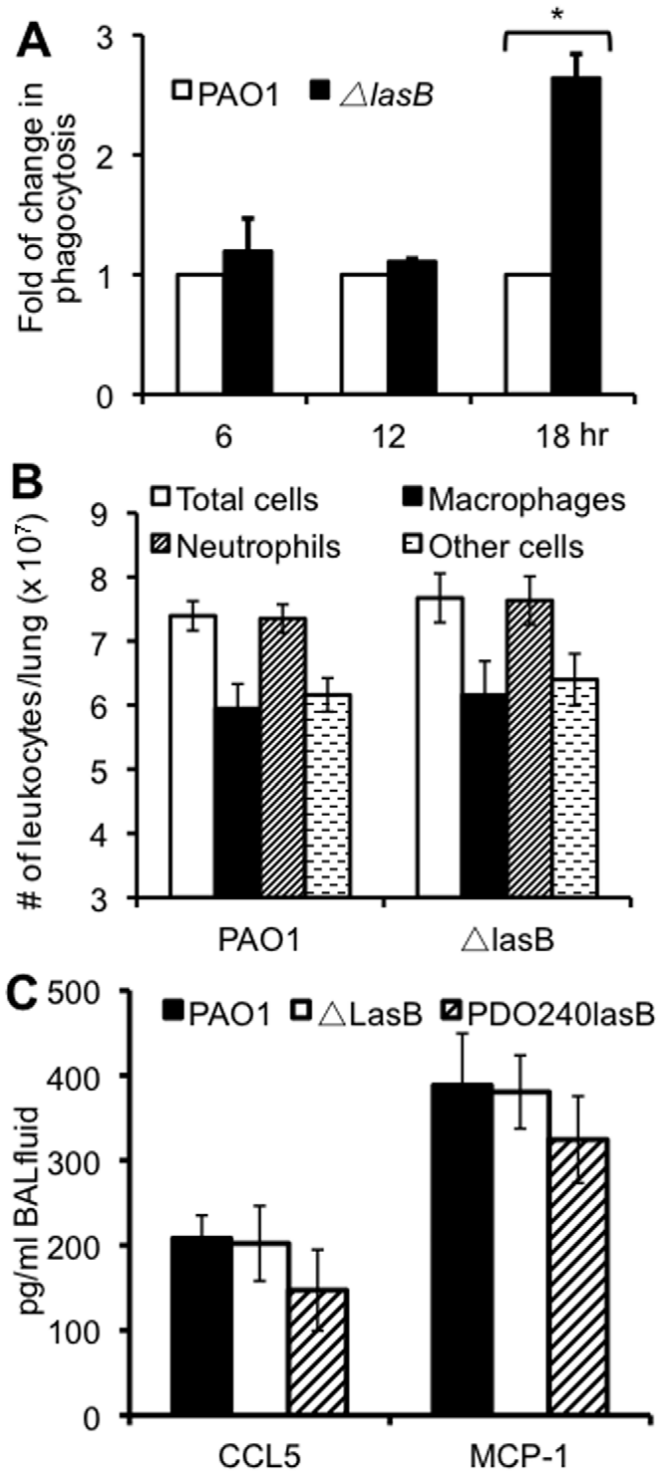
The Δ*lasB* mutant bacteria are more susceptible to SP-A-mediated opsonization *in vivo*. (A) SP-A^+/+^ mice were intranasally infected with 1×10^7^ of wild-type *P. aeruginosa* PAO1 or Δ*lasB* bacteria. At each time interval, infected mice (n = 5) were lavaged for macrophages and infiltrating leukocytes. Cells were centrifuged, washed and the engulfed bacteria were enumerated by gentamicin exclusion assay. Changes in bacterial phagocytosis were calculated based on the number of intracellular PAO1. The mean + standard deviation is shown. *p<0.01 when comparing the number of internalized Δ*lasB* bacteria against PAO1 bacteria. (B) Leukocyte profiles in mouse lungs infected with PAO1 or Δ*lasB* bacteria. Macrophages and neutrophils within BAL fluids were determined using antibody specific against each cell type by using flow cytometry. (C) Profiles of macrophage and neutrophil chemotactic chemokines CCL5 and MCP1 in mouse lungs infected with PAO1, Δ*lasB* or PDO240lasB bacteria.

The *in vitro* phagocytosis results presented in Fig. 6 suggest that proteolytic degradation of SP-A is required to negate enhanced clearance of *P. aeruginosa* by macrophages. We examined this possibility by performing *in vivo* phagocytosis assays. After infection with the wild-type PAO1 or Δ*lasB* bacteria, mouse lungs were lavaged at 6, 12 and 18 hours post infection. Bacteria that were internalized by lung leukocytes within the BAL fluids were enumerated by gentamicin exclusion assays. As shown in Figure 8A, the number of internalized Δ*lasB* bacteria was not different than internalized PAO1 bacteria at 6 and 12 hr post-infection. However, by 18 hr post infection, the number of internalized Δ*lasB* bacteria was 2.6 fold higher than PAO1. The latter time point correlates with the time interval when a significant amount of mSP-A is degraded by PAO1 bacteria (Figure 5C) but not by the Δ*lasB* bacteria. The increase in the phagocytosis of Δ*lasB* bacteria was not due to disproportionately higher levels of professional phagocytes because flow cytometry analyses showed that both PAO1 and Δ*lasB*-infected mouse lungs had similar numbers of neutrophils and macrophages (Figure 8B). Leukocytes analysis was supported by ELISA assays, which indicated that the levels of the neutrophil and macrophage chemotactic chemokines CCL5 and MCP1 were not statistically different between mouse lungs infected with PAO1, Δ*lasB* or PDO240lasB (Figure 8C). These results suggest that the Δ*lasB* bacteria were unable to protect themselves from mSP-A-mediated opsonization *in vivo* due to their inability to remove the collectin through proteolytic degradation.

### Aggregation of Δ*lasB* bacteria in the presence of SP-A

**Figure 9:**
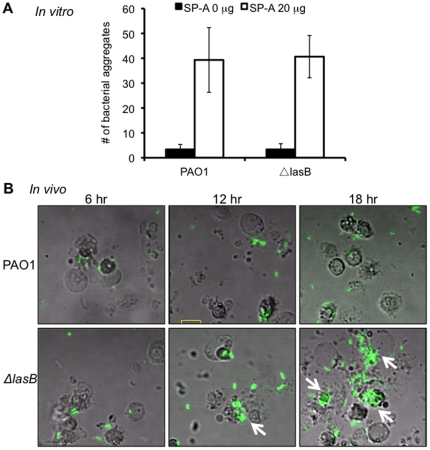
The Δ*lasB* mutant bacteria are more susceptible to SP-A-mediated aggregation *in vivo*. (A) *In vitro* aggregation of GFP-expressing wild-type *P. aeruginosa* PAO1 or Δ*lasB* bacteria co-incubated with hSP-A and observed under fluorescent microscopy. (B) *In vivo* aggregation of GFP-expressing wild-type *P. aeruginosa* PAO1 or Δ*lasB* (arrows) bacteria lavaged from mouse lungs 18-hr post-infection (n = 5) observed under FLUOVIEW FV300 confocal microscope.

SP-A aggregates microbes, which are phagocytized at higher efficiency by professional phagocytes [2], [3], [7], [16]. We used fluorescent microscopy to examine whether there was a difference in the efficiency of SP-A-mediated aggregation of GFP-expressing PAO1 versus Δ*lasB* bacteria. As shown in Figure 9A, after 120 min of aggregation by hSP-A, the number of Δ*lasB-GFP* aggregates was slightly higher than PAO1-GFP. However, the increase was not statistically significant. This is not surprising considering that excess amounts of intact hSP-A still present in the mixture (Figure 4A). Also, we examined the bacterial aggregates in the BAL fluids at 18 hr post-infection (Figure 9B). The Δ*lasB-GFP* bacteria were frequently found in aggregates, suggesting of opsonization by mSP-A (Figure 9B, arrows). In contrast, no aggregates of PAO1-GFP bacteria were apparent in infected mouse lungs. Taken together, these results suggest that failure by the Δ*lasB* bacteria to degrade SP-A allows the collectin to effectively aggregate, opsonize and facilitate the phagocytosis and preferential clearance of the LasB-deficient bacteria.

### Δ*lasB* bacteria are attenuated in degradation of pulmonary innate immunity protein lysozyme

**Figure 10:**
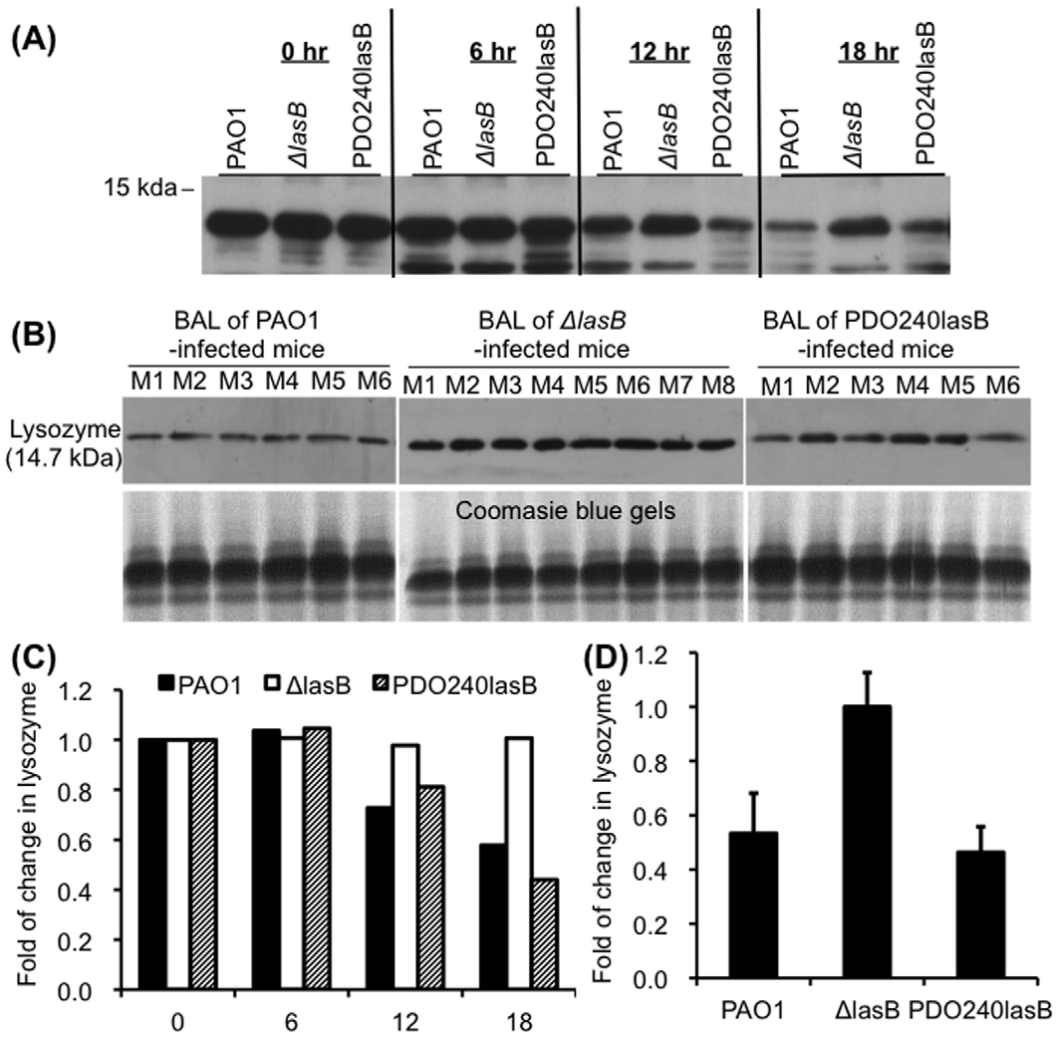
Lysozyme-degrading ability is reduced inΔ*lasB* mutant bacteria *in vitro* and in *vivo*. (A) Chicken lysozyme (5 µg) was incubated with 1×10^8^ PAO1, Δ*lasB* or PDO240LasB bacteria for the indicated time intervals. Lysozyme degradation was assessed by western blot analyses using 10 µl of lysozyme/bacterial suspension. (B) Elastase deficient Δ*lasB* mutant is attenuated in the degradation of mouse lysozyme during lung infection. BAL fluids from Fig. 5C were used for western blot analyses. (C) Densitometry quantification of chicken lysozyme degradation in A. (D) Densitometry analysis of lysozyme degradation by PAO1, Δ*lasB* and PDO240lasB in mouse lungs 18 hr post-infection. The amounts of remaining mouse lysozyme in Δ*lasB* were set to the value of 100%. *p<0.05 when compared with the amount of lysozyme in BAL fluids from lungs infected with PAO1 or PDO240lasB against BAL fluids from Δ*lasB*-infected mice.

Our phagocytosis assays shown above have demonstrated that SP-A enhances the phagocytosis of *P. aeruginosa* by ∼2-3 fold. However, the final difference in bacterial load of SP-A^+/+^ versus SP-A^-/-^ is ∼ 100 fold (Figure 2A), suggests that LasB may be required to degrade other components of pulmonary antimicrobial proteins. We examined whether the Δ*lasB* bacteria are attenuated in degradation of lysozyme, which has been previously shown to be important against *P. aeruginosa* [57]. In addition, we have previously shown that SP-A and lysozyme act synergistically to permeabilize the membranes of wild-type *P. aeruginosa* strain PAO1 [22]. Given this unanticipated discrepancy, we examined *in vitro* and in BAL fluids of infected mouse lungs for evidence of reduced degradation of lysozyme. As shown in Figure 10, LasB was able to degrade lysozyme both *in vitro* and *in vivo* experimental conditions. To confirm lysozyme degradation, we incubated 5 µg/ml lysozyme with 1×10^8^ PAO1, Δ*lasB,* and PDO240lasB bacteria. After 18 hr incubation, lysozyme exposed to Δ*lasB* mutant remained intact (Figure 10A). In contrast, PAO1 or PDO240lasB bacteria were able to degrade lysozyme (Figure 10A). Similarly, BAL samples from mice infected with PAO1 or PDO240lasB had reduced amounts of lysozyme (Figure 10B). In contrast, BAL samples from mice infected with Δ*lasB* mutant still contained intact lysozyme. Densitometry quantifications indicated that by 18 hr, PAO1 and PDO240lasB had degraded 50-60% more lysozyme than the Δ*lasB* mutant *in vitro* (Figure 10C) and *in vivo* (Figure 10D). Thus, infection by *P. aeruginosa* likely induced the expression of lysozyme, which was subsequently degraded by LasB and other exoproteases produced by PAO1 or PDO240lasB. In contrast, due to inability of the Δ*lasB* mutant to elaborate adequate exoprotease activity, lysozyme remained intact.

## Discussion

*P. aeruginosa* LasB is an important virulence factor during host infections. In addition to damaging tissues and disrupting intercellular junctions of lung epithelia, LasB also is capable of degrading components of the innate and acquired immune system, including cytokines and chemokines, antimicrobial peptides, immunoglobulins, serum complement factors, and surfactant protein [31], [40], [43]–[50], [58], [59]. However, most of these studies were performed *in vitro* with a combination of purified elastase and purified host components, or purified host component exposed to *P. aeruginosa*. Thus, direct proof of LasB-mediated proteolysis in lung infection is lacking. In this study, we provide evidence that *P. aeruginosa* elastase reduces the phagocytosis of the bacteria in mouse lungs by degrading SP-A, an important innate immune system component that opsonizes and membrane permeabilizes microbes. By comparing lung infections between SP-A^+/+^ and SP-A^-/-^ mice using a combination of wild type *P. aeruginosa* strain PAO1 and isogenic mutant strain Δ*lasB*, we demonstrate that: (i) the Δ*lasB* mutant is attenuated in the lungs of SP-A^+/+^ mice but is fully virulent in the lungs SP-A^-/-^ mice; (ii) inability to secrete LasB impairs the ability of *P. aeruginosa* to degrade SP-A both *in vitro* and in mouse lungs; (iii) LasB deficiency does not result in increased susceptibility of *P. aeruginosa* to membrane permeabilization by SP-A; (iv) failure to degrade mSP-A results in increased opsonization and enhanced clearance of the Δ*lasB* mutant from the lungs of SP-A^+/+^ mice; (v) substantial amounts of SP-A degradation by LasB needs to occur before the phagocytosis of *P. aeruginosa* by professional phagocytes is significantly reduced. Collectively, these results suggest that LasB affords a protective role to *P. aeruginosa* by negating the ability of SP-A to serve as an opsonin that helps to augment phagocytosis.

*In vitro* degradation of hSP-A by exoproteases of *P. aeruginosa* was previously reported [43], [52]. These authors observed the degradation of hSP-A when the collectin was co-cultured with *P. aeruginosa,* and with BAL fluids from the lungs of CF patients chronically colonized by the bacterial pathogen. After purification and mass spectroscopy analysis, the proteolytic enzyme was identified as *P. aeruginosa* elastase. By comparing the infection of SP-A^+/+^ versus SP-A^-/-^ mouse lungs using both wild-type PAO1 and the Δ*lasB* mutant, we reveal that LasB plays an important role in negating the innate immunity role of mSP-A through proteolytic degradation of the collectin.

Apart from serving as an opsonin, SP-A also has the ability to permeabilize microbial membranes, similar to antimicrobial peptides [22], [23], [26], [27], [58], [60]. It has been suggested that SP-A may be one of the major lung innate immunity proteins that permeabilize bacterial membranes [53]. However, we have reported that wild-type *P. aeruginosa* is resistant to SP-A-mediated membrane permeabilization [26], [27]. *P. aeruginosa* confers resistance to SP-A-mediated membrane permeabilization by elaborating LPS, flagella, phosphoenolpyruvate phosphotransferase and salicylate biosynthesis, and exoproteases [22], [26], [27]. Especially interesting is that the loss of flagella seems to reduce the ability of *P. aeruginosa* to synthesize adequate LPS, resulting in increased susceptibility to SP-A. Furthermore, flagella-deficiency also causes *P. aeruginosa* to produce less exoproteases [22]. As we have shown here, the loss of LasB, a major exoprotease in *P. aeruginosa*, renders the pathogen susceptible to increased clearance from lungs through opsonization, not membrane permeabilization. However, we have previously shown that the flagella-deficient mutants of *P. aeruginosa* do not exhibit increased susceptibility to SP-A-mediated opsonization. This discrepancy could be explained because the *in vitro* and *in vivo* phagocytosis studies of the flagella mutants were performed for only 60 - 120 minutes [26], and the data is similar to what we have observed for the Δ*lasB* mutant, where phagocytosis was carried out for 60 min (Fig. 5). However, as we have demonstrated, wild-type *P. aeruginosa* PAO1 induces a time-dependent degradation of SP-A with a corresponding reduction in SP-A-mediated opsonization at 6-hr or longer post-incubation *in vitro*, or 18 hr *in vivo*. In contrast, the Δ*lasB* mutant bacteria are unable to degrade adequate amounts of SP-A, and are increasingly cleared by hSP-A-augmented phagocytosis by RAW 246.7 macrophages through the 18 hr incubation. We are currently performing experiments to clarify the relationship between exoprotease deficiency of flagella mutants and susceptibility to SP-A-mediated opsonization.

Our comparative *in vivo* phagocytosis assays indicate that the differences between the number of PAO1 and Δ*lasB* bacteria internalized by pulmonary leukocytes are only apparent 18 hr post-infection, but not at earlier time points. This observation is reflective of the amounts of intact mSP-A remaining in the infected lungs, which are not substantially degraded until 18 hr post-infection. These results suggest that the kinetics of mSP-A degradation are slower during lung infection. This is not surprising considering the complexity of the pulmonary immune response during an acute pneumonia infection. For example, it is known that neutrophil elastase also degrades SP-A [60], [61]. Thus, at 18 hr post-*P. aeruginosa* infection when the neutrophil influx is prominent (Fig. 7B), it is possible that a combination of LasB, other minor *P. aeruginosa* exoproteases and neutrophil elastase all combine to afford a quantifiable difference in mSP-A degradation to result in an alteration in the phagocytosis of PAO1 and Δ*lasB*. However, the contribution of neutrophil elastase seems less likely because infections by both PAO1 and Δ*lasB* result in similar leukocytic infiltration. In addition, the loss of LasB function should trigger *P. aeruginosa* to overproduce other exoproteases to compensate for the loss of the former, or at least maintain the secretion of these exoproteases at the wild-type levels.

It is known that *P. aeruginosa* has a propensity to reduce the expression of many virulence factors such as elastase, lipase, exotoxin A, etc., during chronic infection of CF airways [33]. In contrast, many of these clinical CF isolates overproduced alginate, a major polysaccharide capsule, resulting in a mucoid phenotype. Mucoid *P. aeruginosa* are more resistant to phagocytosis. Previously, it was shown that LasB plays a role in the biosynthesis of alginate [62]. Overexpression of LasB in both mucoid and non-mucoid *P. aeruginosa* cells, stimulates alginate synthesis [62]. Mechanistically, this is achieved by a genetic rearrangement that triggers mucoidy in *P. aeruginosa,* which also allows retention of elastase in the periplasm in an active oligomeric form. The LasB cleaves the 16 kDa form of nucleoside diphosphate kinase (Ndk) to a truncated 12 kDa form. Processed NdK is important for the generation of GTP required for alginate synthesis [62]. Thus, the loss of LasB may negatively affect alginate production, resulting in increased susceptibility to SP-A-mediated opsonization. Even though we cannot rule out this possibility, we predict that the effect of alginate is minimal since it is only present in limited amounts in non-mucoid *P. aeruginosa*.

Collectins, including SP-A, frequently bind and aggregate microbes. Aggregated microorganisms are phagocytized at higher efficiency [2], [3], [7], [16]. van Rozendaal et al reported that SP-D inhibits protein synthesis and hyphal outgrowth in *Candida albicans* [63]. These authors speculated that inhibition of protein synthesis was an indirect consequence of fungal aggregation restricting access of the organisms to essential nutrients. Undoubtedly, aggregation of Δ*lasB* mutant bacteria but not wild-type PAO1 at late stages of infection promotes more efficient clearance of the former. We are currently determining whether aggregation of Δ*lasB* bacteria is limiting access to nutrients.

One unresolved issue regarding our study is the relative contribution of SP-A versus other pulmonary innate immunity proteins in controlling *P. aeruginosa* infection. As we have discussed, exposure to SP-A increases the phagocytosis of *P. aeruginosa* by 2-3 fold, and that at late stages of acute pneumonia infection, the Δ*lasB* mutant bacteria are phagocytized better than the wild-type PAO1 because of the latter’s ability to degrade mSP-A. However, it was likely that a 2-3 fold increase in phagocytosis would not have accounted for ∼100 fold increase in the clearance of Δ*lasB* mutant bacteria. ELISA assays indicated that the levels of neutrophil and macrophage chemotactic chemokines CCL5 and MCP1 in the mouse lungs infected by PAO1 versus Δ*lasB* were not significant different, suggesting that these chemokines were not susceptible to degradation by LasB. However, additional experiments suggest that LasB is also a major exoprotease that degrades lysozyme, which is known to have antimicrobial activities [57]. Thus, we cannot rule out that a synergistic or additive role of various pulmonary innate immunity proteins, which are susceptible to LasB degradation, may have contributed to removal of Δ*lasB* mutant bacteria. We are currently examining in detail the susceptibility of these pulmonary innate immunity proteins to LasB.

In conclusion, our study demonstrates that Δ*lasB* mutant is unable to degrade mSP-A. This leads to more efficient clearance by SP-A-mediated opsonization in infected mouse lungs. Therapeutic strategies aiming at inactivating the activity of this exoprotease may enhance the clearance of *P. aeruginosa*, and reduce the morbidity and mortality during lung infections mediated by this versatile pathogen.

## Materials and Methods

### Chemicals

All chemicals were purchased from Sigma Chemical Co. (St. Louis, MO), unless stated otherwise.

### Bacterial strains, media and growth conditions

The parental wild-type *P. aeruginosa* PAO1 strain was originally obtained from Dr. Michael Vasil as previously described [22], [27], [64]. The LasB-deficient mutant PDO240 (Δ*lasB*) was derived by gene replacement by McIver et al [65] in the same PAO1 strain. The genetically-complemented strain PDO240LasB was derived by transforming the Δ*lasB* mutant with the plasmid pKSM3 carrying a copy of the wild-type *lasB* gene [38]. Bacterial strains were grown in Luria-Bertani Broth (LB) for 16 hr at 37°C, resuspended in LB with 20% glycerol and frozen in aliquots at -80°C. Before each experiment, bacteria were cultured from frozen stocks in LB with or without antibiotics to stationary phase (OD600nm ≈ 3.0). Bacterial density was determined spectrophotometrically and was correlated with numbers of viable bacteria by colony-forming units (cfu) after plating serial dilutions on agar plates. When required, antibiotics were used at the following concentrations: for *P. aeruginosa*, carbenicillin (300 µg/ml), gentamicin (30 µg/ml), spectinomycin (100 µg/ml), tetracycline (60 µg/ml); for *Escherichia coli* DH5α (66), carbenicillin (100 µg/ml) and tetracyclin (20 µg/ml).

### Murine macrophage cell line

Murine RAW 264.7 macrophages (ATCC #TIB-71) were maintained in DMEM supplemented with 10% FBS, and 1% streptomycin and penicillin, respectively, at 37°C in the presence of 5% CO_2_.

### Purification of human SP-A

Human SP-A was purified from the lung washings of patients with alveolar proteinosis as previously described [67]. Pure hSP-A samples were stored in membrane permeabilization buffer (5 mM Tris, 150 mM NaCl, pH 7.4)^ ^at -20°C. The preparations were deemed free of EDTA by a modified spectrophotometric assay, using ß-phenanthrolene–disulfonic acid as the indicator [68].

### Protein assays

Protein concentrations were routinely determined by the bicinchoninic acid protein assay kit (BCA; Pierce Chemical Co., Rockford, IL, USA), using bovine serum albumin (BSA) as a standard. Protein samples were resolved on 8–16% SDS-PAGE gel and stained with Coomassie blue or silver nitrate.

### Animal husbandry

Swiss Black SP-A^-/-^ mice, a gift of J. Whitsett/T. Korfhagen, were derived from embryonic stem cells after disruption of the mouse SP-A gene by homologous recombination and were maintained by breeding with Swiss Black mice [69]. The SP-A null allele was backrossed into the C3H/HeN genetic background through nine generations [25]. C3H/HeN control (SP-A^+/+^) mice were purchased from Harlan Laboratory (South Easton, MA). All comparisons made with the SP-A^-/-^ mice were with age- and strain-matched C3H/HeN controls. All animals were housed in positively ventilated microisolator cages with automatic recirculating water located in a room with laminar, high efficiency particulate-filtered air. The animals received autoclaved food, water, and bedding. Mice were handled in accordance with approved protocols through the Institutional Animal Care and Use Committee at the University of Illinois at Urbana-Champaign.

### Mouse infection

Single intranasal infections of SP-A^+/+^ and SP-A^-/-^ mice (groups of 4-8) were performed with 1×10^7^ of PAO1, Δ*lasB* or PDO240LasB bacteria as we have previously published [22], [26], [27]. After 18 hr, mouse lungs (n = 5) were harvested for bacterial enumeration, or broncho-alveolar lavaged (BAL) for proteins used in western blots or membrane permeabilization analyses (n = 5-8). Virulence attenuation was defined as the log_10_ difference in CFU of various *P. aeruginosa* bacteria recovered from the lung tissues of SP-A^+/+^ versus SP-A^-/-^ mice.

### BAL

BAL was performed on *P. aeruginosa*-infected mice (n = 5) as we have previously described [22], [27]. The trachea was exposed and intubated with a 1.7-mm outer diameter polyethylene catheter. BAL was performed by instilling PBS in 3×1 ml aliquots per mouse. In some experiments, the BAL samples were pooled for membrane permeabilization assays.

### Flow cytometry of mouse lung leukocytes

BAL fluids from *P. aeruginosa*-infected mice (n = 5) were centrifuged and resuspended in flow cytometry staining buffer. Cells were pre-incubated with anti-mouse CD16/CD32 (cat#: 14-0161, eBioscience, San Diego, CA) for 20 minutes on ice prior to staining to block non-specific Fc-mediated interactions. Mouse macrophages were labeled with primary antibody anti-mouse F4/80-PE (cat#: 12-4801-80, eBioscience). Mouse neutrophils were labeled with anti-mouse Ly-6G-FITC (cat#: 11-5931-81, eBioscience). Flow cytometric acquisition was performed using a C6 flow cytometer (Accuri, Ann Arbor, MI) and analyzed with CFlow Plus version 1.0.

### Membrane permeabilization assays

The effect of SP-A on the cell membrane integrity of *P. aeruginosa* and *E. coli* DH5α was assessed by determining permeability to a phosphatase substrate, Enzyme-Labeled Fluorescence 97 (ELF-97) (Molecular Probes, Carlsbad, CA), as we have previously described [22], [26], [27]. hSP-A (50 µg/ml) or mouse BAL fluids (50 µg total protein) was incubated with 1×10^8^ stationary phase *P. aeruginosa* or *E. coli* bacteria/ml in 100 µl of membrane permeabilization buffer for 15 min at 37°C, and 100 µM ELF97 phosphatase substrate was added. Fluorescence was measured at excitation and emission wavelengths of 355 and 535 nm, respectively, for 90 - 120 min.

### Exoprotease assays

Exoprotease activities were determined by the Sensolyte^TM^ Red Protease Assay Kit (AnaSpec Inc, San Jose, CA, Cat # 71140) using cell-free supernatants of stationary phase cultures from *P. aeruginosa* PAO1, Δ*lasB* or PDO240LasB grown in LB.

### *In vitro* hSP-A and lysozyme degradation assays

*P. aeruginosa* strains PAO1, Δ*lasB* or PDO240LasB bacteria were cultured in LB overnight to late stationary phase. hSP-A (25 µg) or chicken lysozyme (5 µg) was added to 1×10^8^ *P. aeruginosa* cells resuspended in 250 µl of fresh LB supplemented with 2 mM CaCl_2_ in the presence or absence of 0.6 mM ZnCl_2_. At indicated time intervals, a 10 µl aliquot of each bacterial-SPA mixture or cell-free supernatants was mixed with loading buffer for SDS-PAGE and Western blot analysis.

### Western blot

Western blot analyses were performed using standard protocols [70]. Briefly, protein samples of hSP-A, mouse BAL fluids, *P. aeruginosa* bacteria or culture supernatants were resolved by SDS-PAGE and electro-blotted onto Immobilon P polyvinylidene difluoride membranes (Millipore, Bedford, MA). The membranes were then incubated for 60 min at room temperature in blocking solution (PBS containing 3% BSA), followed by a 4-hr incubation with polyclonal antibody against hSP-A and mSP-A (Santa Cruz Biotecnology Inc, Santa Cruz, CA), a polyclonal antibody against chicken and mouse lysozymes [57], or with a polyclonal antibody against LasB [38], [39]. The membranes were hybridized with horseradish peroxidase-conjugated goat anti-mouse IgG secondary antibody. Immune complexes were visualized using the ECL Western Blotting Detection System (Amershan Biosciences, Piscataway, NJ) and Kodak BIOMAX (Kodak, Rochester, NY) X-ray films.

### *In vitro* phagocytosis assays

For *in vitro* phagocytosis experiments, approximately 1×10^6^ RAW 264.7 macrophages were plated on 6-well tissue culture plates overnight. Macrophages were exposed to 10^7^ *P. aeruginosa* cells in the presence of intact hSP-A (10–50 µg/ml), or to a mixture of 1×10^7^ *P. aeruginosa* bacteria and (20 µg/ml) hSP-A that had been incubating for 1, 6, 12 or 18 hr. After 1 hr of infection, monolayer cells were washed 3 times with PBS and incubated with DMEM containing 100 µg/ml gentamicin for 1 hr to kill off extracellular bacteria [69]. Cells were washed again to remove gentamicin, and lysed with 0.5% Triton X-100. The intracellular bacteria were serial diluted for cfu enumeration on agar plates.

### *In vivo* phagocytosis assays

For *in vivo* phagocytosis assays, 1×10^7^ *P. aeruginosa* bacteria were intranasally inoculated into the lungs of SP-A^+/+^ mice (n = 5). Infected lungs were BAL at indicated time intervals with PBS to obtain alveolar leukocytes. BAL samples were centrifuged and washed three times with PBS. Leukocytes were treated with DMEM containing 100 µg/ml gentamicin for 1 hr to kill off extracellular bacteria. The bacteria that were internalized by phagocytes were enumerated using the gentamicin exclusion assays.

### Bacterial aggregation assays

The susceptibility of the wild-type *P. aeruginosa* PAO1 and Δ*lasB* mutant bacteria to aggregation by SP-A was assessed by aggregation assay. Briefly, 1×10^7^ stationary phase *P. aeruginosa* bacteria transformed with pUC19-GFP were incubated with hSP-A (20 µg/ml) in 500 µl of DMEM medium supplemented with 2 mM CaCl_2_. The mixtures were rotated at 37°C for 60 min at 120 rpm. Each mixture (10 µl) was spotted on slides and observed under LEICA DMI4000 fluorescent microscope. The number of bacterial clusters was enumerated from 10 independent fields under 10x magnification, in three independent experiments. For *ex vivo* aggregation, mouse lungs of SP-A^+/+^ mice (n = 5) infected with 1×10^7^ wild-type *P. aeruginosa* PAO1-GFP or PDO240-GFP (Table 1) were lavaged at 18 hr post infection. Bacterial aggregates within BAL fluids were observed directly under FLUOVIEW FV300 confocal microscope.

### ELISA assays

Protein levels of chemokines CCL5 and MCP1 in BAL or lung homogenates were determined by ELISA according to the manufacturer’s protocols (Invitrogen, Carlsbad, CA).

### Statistical analyses

Statistical analysis was performed using the Student's *t*-test and one-way analyses of variance (ANOVA). A significant difference was considered to be *p*<0.05.

## Supporting Information

Figure S1 **SP-A-degrading ability is reduced in** Δ** ***lasB*** **mutant bacteria** ***in vitro*** **.** (A) hSP-A (25 µg) was incubated with 1×10^8^ PAO1, Δ*lasB* or PDO240LasB bacteria in LB supplemented with 0.6 mM ZnCl_2_ for the indicated time intervals. hSP-A degradation was assessed by western blot analyses using 10 µl of SP-A/bacterial suspension. Image from one of the three independent experiments is shown. (B) hSP-A degradation *P. aeruginosa* strains in the absence of ZnCl_2_. Immunoblots were probed with anti-SP-A antibody.
(TIF)
