# Supplementary figures and images for: Pseudomonas aeruginosa Elastase Provides an Escape from Phagocytosis by Degrading the Pulmonary Surfactant Protein-A
Source: PLoS One. 2011 Nov 1;6(11):e27091. doi: 10.1371/journal.pone.0027091 (PMC3206073; doi:10.1371/journal.pone.0027091)

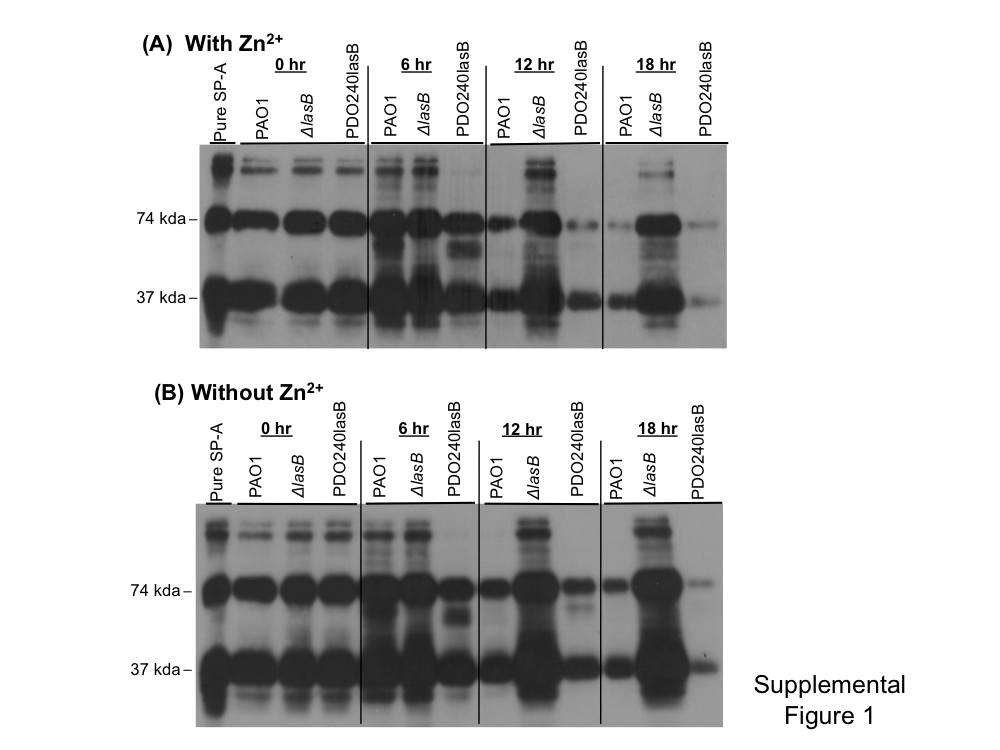

Supplement: Figure S1 — SP-A-degrading ability is reduced in Δ lasB mutant bacteria in vitro . (A) hSP-A (25 µg) was incubated with 1×10^8^ PAO1, ΔlasB or PDO240LasB bacteria in LB supplemented with 0.6 mM ZnCl2 for the indicated time intervals. hSP-A degradation was assessed by western blot analyses using 10 µl of SP-A/bacterial suspension. Image from one of the three independent experiments is shown. (B) hSP-A degradation P. aeruginosa strains in the absence of ZnCl2. Immunoblots were probed with anti-SP-A antibody. (TIF) [file pone.0027091.s001.tif]
